# Pharmaceutical toxicity is a common pattern of inpatient acute poisonings in Birjand City, East of Iran

**DOI:** 10.1038/s41598-023-28353-1

**Published:** 2023-01-24

**Authors:** Kobra Naseri, Zahra Kiani, Zakieh Sadat Sajadi, Omid Mehrpour, Seyed Yoosef Javadmoosavi, Fatemeh Forouzanfar, Mahmood Sadeghi

**Affiliations:** 1 Medical Toxicology and Drug Abuse Research Center (MTDRC), Birjand University of Medical Sciences, Birjand, Iran; 2 Rocky Mountain Poison and Drug Safety, Denver Health, Denver, CO USA; 3 Social Determinants of Health Research Center, Birjand University of Medical Sciences, Birjand, Iran; 4 Neuroscience Research Center, Mashhad University of Medical Sciences, Mashhad, Iran; 5 Department of Neuroscience, Faculty of Medicine, Mashhad University of Medical Sciences, Mashhad, Iran

**Keywords:** Diseases, Health care, Medical research, Signs and symptoms

## Abstract

Information on the pattern of acute poisonings in hospitals of Birjand city, Iran, is limited. This study aimed to address this knowledge gap by examining the admissions in a major poisoning center in eastern Iran. This cross-sectional study included patients admitted to the Imam Reza Hospital in Birjand over 12 months. Medical records of the poisoned patients were reviewed, and the study variables were used for data analysis. During the study period, 534 cases of acute poisonings were evaluated. The patient's ages ranged from 12 to 84 years, with a high rate of poisonings between 15 and 35 years. The female predominance in poisoning cases was 52.1%. Most cases of poisonings occurred in spring, and the common route of exposure was oral (93.1%). The incidence of poisoning in married couples, uneducated patients, and residents of urban areas was 56.5%, 90.1%, and 74.6%, respectively. Patients with a previous medical history experienced addiction and psychiatric disorders. Intentional poisoning accounted for 23.4% of acute poisoning cases referred to the hospital in the current study. The main groups of toxicants were pharmaceutical products (48.1%), narcotics (25.8%), chemical products (10.1%), envenomation (7.1%), and alcohol (1.7%). The mean hospital stay was 2.5 ± 3.0 days, and the final treatment outcome was complete recovery, except for one patient intoxicated by warfarin and alprazolam. Our results indicate that the high toxicity cases were related to pharmaceutical product and opioids abuse, especially methadone (8.4%), alprazolam (7.9%), clonazepam (7.5%), and acetaminophen (9.9%) taken orally and more commonly happened at home. Due to the high rate of deliberate poisonings, especially among young adults and students, monitoring drug distribution and exceptional attention to mental health should be seriously considered by national health authorities to prevent suicide attempts.

## Introduction

Acute poisoning is a significant public health problem and is a frequent reason for referral to emergency departments^1^. Poisoning can be intentional or unintentional^2^. Accidental poisonings remain a common cause of intoxication in children. While accidental poisoning is more common in pediatrics, deliberate self-poisoning occurs more frequently in adults^3^. Unintentional poisonings include food poisoning, chemical and pharmaceutical intoxication, envenomation, and pesticide toxicity^4^.

At least 500,000 people die annually from poisoning worldwide^5^. Poisoning following chemical or pharmaceutical products is a common cause of emergency admission in Iran, making up 2.5–5% of all deaths^6^. Various factors influence the pattern of poisonings. Advances in technology, agriculture, and pharmacology have led to significant changes in the pattern of poisonings. Household chemicals and prescription drugs are the most common cause of poisoning in developed countries^7^. Analysis of the pattern of poisonings in a specific area will play an essential role in identifying risk factors^8^. Non-drug poisonings are also common in many parts of the world. Organophosphorus compounds, aluminum phosphide, opium, crack and detergents are examples of non-drug poisonings^9,10^. Since most poisoning cases remain unreported, the real number of incidences could be much higher^11^.

Most poisonings in Iran are intentional and occur predominantly in the young population. Moreover, opioids and insecticides are accounted for the most important causes of death^12^. The pattern of poisonings in different parts of the world may differ. Pesticides and snakebites are main causes of poisoning in the region in the east of Africa^13^. Pesticides were mentioned as a common source of adult poisoning in South India. Most deaths in children due to plant toxins and paraffin have raised concerns^14^. In 2003, sedative-hypnotic drugs and opioids developed a pattern of acute poisoning in Tehran, Iran. The reported mortality rate was 1.3%^12^.

Knowing the pattern of poisonings can help prevent toxicities, especially by reducing the incidence of suicide. In different countries, many studies are conducted annually to evaluate the pattern of poisonings and associated parameters such as toxicant type, cause of poisoning and the outcome. However, very few studies show the pattern of poisoning in South Khorasan in the east of Iran. Therefore, we aimed to assess the pattern of acute poisoning in all poisoned patients admitted to the Imam Reza hospital in Birjand City, Iran. Moreover, it was aimed to compare our observed pattern of acute poisoning and drug overdose with the literature we reviewed in this regard.

## Materials and methods

A descriptive cross-sectional study was conducted to investigate the epidemiological pattern of acute poisoning in cases admitted to the Imam Reza Hospital in Birjand City, east of Iran. Data were collected retrospectively. Imam Reza Hospital, one of the oldest hospitals in the city, affiliated with Birjand University of Medical Sciences, is a teaching hospital and is the only referral center specialized for toxicology patients in the South Khorasan Province. The city had a population of 200,000 in 2016. Imam Reza Hospital is the province's most important emergency and accident center. In addition to poisoning and emergency departments, the setting has other departments such as psychiatry, trauma, surgery, and urology. All patients referred to the emergency department of Imam Reza Hospital and other hospitals in the province who were diagnosed with acute poisoning and needed to be hospitalized were referred to the poisoning department of Imam Reza Hospital.

In this research, the study population was included in the study for one year. All patients diagnosed with acute poisoning hospitalized during this year were included in the study. Information on inpatients' acute poisonings, such as age, gender, and cause of poisoning, was collected from the medical records, which were accurately completed. The inclusion criteria were male and female patients of any age and chief complaints of acute poisoning. The exclusion criteria included a presentation with burns, trauma, etc. Although 897 patients met the inclusion criteria, 363 patients were excluded from the study due to incomplete medical records. Included cases that met the criteria of our study (534) were analyzed.

Whole blood, serum, and urine samples were sent to the laboratory for toxicological analysis. As the first step, screening tests are widely used to identify drugs and poisons in samples. For positive results, confirmatory testing is performed. UV/visible spectrophotometry methods are applicable to determine the level of acetaminophen, salicylate and cholinesterase enzyme activity.

The studied variables included age, gender, cause of poisoning, route of exposure, toxicant type, season, marital status, levels of education, job, location of poisoning, the time gap between poison intake and hospitalization, past medical history, treatment duration and outcome. The cause of poisoning was used to categorize accidental or suicidal poisoning. The distinction between suicidal and accidental administration was made according to the history of the referred patients at admission. Route of exposure indicates whether the exposure was oral, inhalation or envenomation. Moreover, pharmaceutical products, envenomation, narcotics, alcohol, or chemical products determine the toxicant type. Last but not least, the type of access to the toxicant refers to whether it was obtained from home or a pharmacy.

A standardized data collection sheet was used based on the variables mentioned above. A prepared questionnaire was filled in for each patient to collect demographic characteristics. Gathered data were statistically analyzed using SPSS software version 21, and a p-value < 0.05 was considered a significant level. Results were presented as descriptive statistics (frequency distribution, percentage) and quantitative analysis (mean ± SD, statistical tests). The Kolmogorov–Smirnov Test was used to determine the normal distribution of quantitative variables. The Independent Samples T Test or the Student’s T-Test was used to compare two groups whose means were not dependent. The Mann–Whitney Test was used to compare two groups when the variable was not normally distributed. Qualitative variables were compared to statistical analysis using Chi-square (X^2^). Informed consent was obtained from the patients for using their data in this study. Confidentiality was maintained at all times regarding the collected information. The input of names into the statistical software was not performed. Instead, a previously allocated number was selected for each patient. The ethics committee approved the study of Birjand University of Medical Sciences (ethics code IR.BUMS.REC.1396.143). Research and all methods were conducted following the Declaration of Helsinki and guidelines on Good Clinical Practice.

## Results

**Figure 1:**
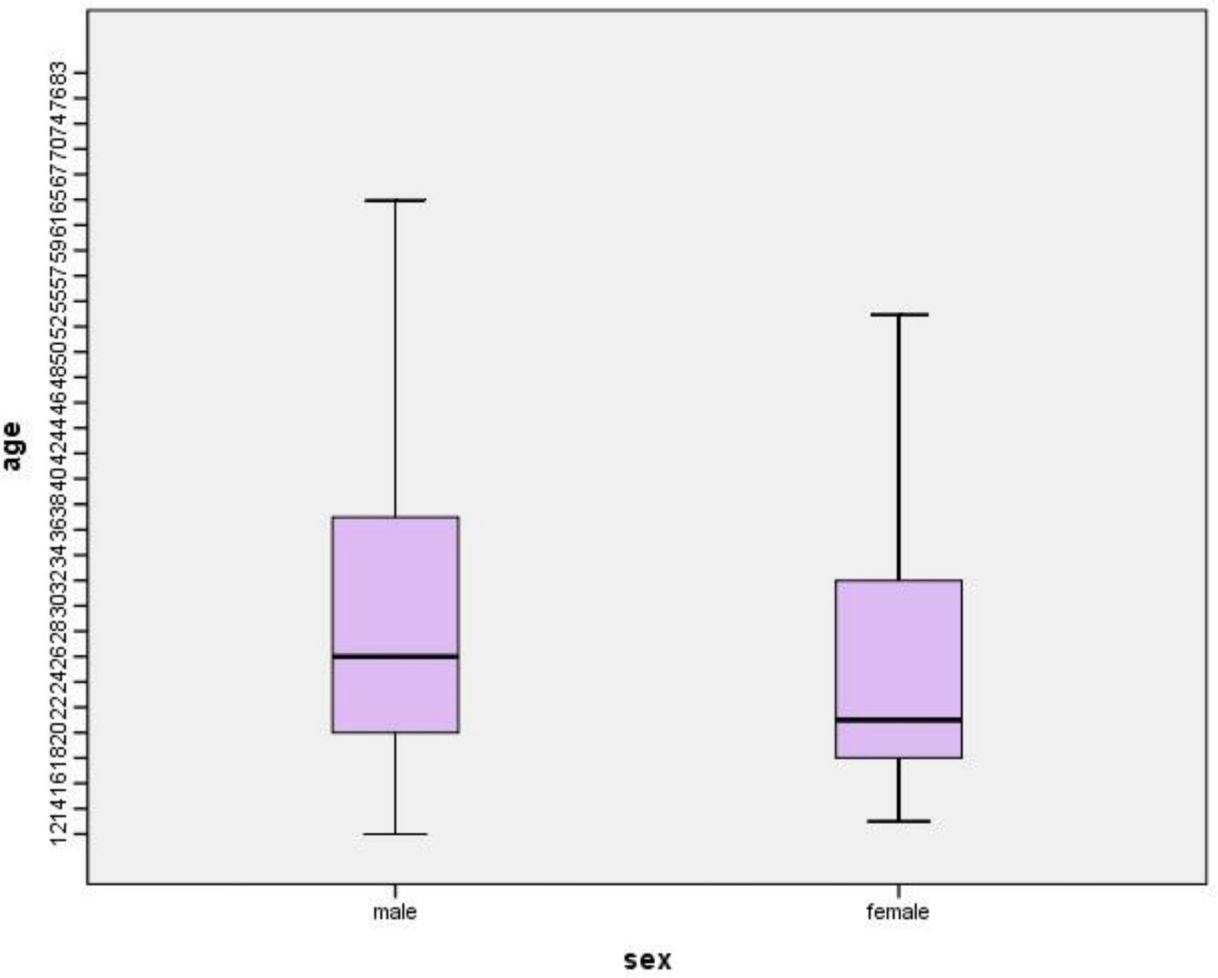
Age of subjects studied by gender.

During the study period, 534 medical records were evaluated for the incidence of acute poisoning. The patient's ages ranged from 12 to 84 years, and 381 patients (71.3%) were between 15 and 35. The mean age of inpatients with acute poisoning was 28.7 ± 14.2 years. Among patients, 52.1% were females, and the rest (47.9%) were males. The frequency distribution of the studied population’ age and the other demographic variables are shown in Tables 1 and 2. The age of incidence of poisoning was different in men and women (p-value < 0.001). The age of male (31.0 ± 14.7 years) poisoned patients was higher than that of female (26.6 ± 13.3 years) patients (95% CI 2.08–6.87). A comparison of the distribution of different ages in each gender is shown in Fig. 1. Most cases of poisonings occurred in spring (32.6%). Cases of poisoning related to the other seasons are 20.0% in summer, 18.2% in autumn, and 29.2% in winter. 74.6% of the patients lived in the city, and the rest (25.4%) were in the countryside. Concerning marital status, 43.5% were single, and 56.5% were married. The frequency distribution of jobs in the poisoned population was as below: 55.0% had a job, 25.3% were students, 4.7% were soldiers and 15.0% were unemployed. Moreover, 9.9% possessed university degrees, while 90.1% did not. Thirty-four patients were not hospitalized and were released from the hospital after a few hours of stay while being under clinical supervision. Hospital policies for poisoned patients are to remain in observation for at least one night. However, 34 patients insisted on not being hospitalized, and therefore were discharged from the emergency department with their consent. We indeed examined the data of those patients in our study. 34 patients admitted to an Intensive Care Unit (6.4% of patients).

**Table 1:** Description of poisoned patients of Imam Reza Hospital, Birjand, September 2017 to August 2018 (n = 534).

Variable	Frequency	Percentage
Age (years)		
< 15	31	5.8
15–35	381	71.3
35–50	71	13.3
50–65	30	5.6
> 65	21	3.9
Gender		
Female	278	52.1
Male	256	47.9
Cause of poisoning		
Suicidal	125	23.4
Accidental	409	76.6
Route of exposure		
Oral	497	93.1
Envenomation	36	6.7
Inhalation	1	0.2
Place of residence		
Urban	398	74.6
Rural	136	25.4
Toxicant type		
Pharmaceutical products	257	48.1
Envenomation	36	6.7
Narcotics	138	25.8
Alcohol	9	1.7
Chemical products	54	10.1
Unknown	40	7.5
Outcome		
Recovery/discharge	533	99.81
Death	1	0.19
Total	534	100.0

**Table 2:** Frequency distribution of age and treatment duration of inpatients' acute poisonings.

	N	Minimum	Maximum	Mean	Std. deviation
Age (year)	534	12	84	28.7	14.2
Age (year) by gender					
Male	256	12	84	31.0^a^	14.7
Female	278	13	80	26.6	13.3
Treatment duration (day)	534	1	30	2.5	3.0
Treatment duration (day) by gender					
Male	256	1	21	2.6^b^	3.1
Female	278	1	30	2.4	2.9

^a^p < 0.001 (Z = − 4.786).

^b^p = 0. 679 (Z = − 0. 414).

Overall, the mean hospital stay was 2.5 ± 3.0 days and ranged from less than one day to 30 days. Most patients (88.6%) needed less than four days of hospitalization. There was no difference (p = 0.679) between the mean of treatment duration among men (2.6 ± 3.1 days) and women (2.4 ± 2.9 days) in this study. Within three hours of intake, 58.4% of patients were admitted to the hospital, while 41.6% were referred after three hours of exposure. According to the medical records, the causes of intoxication were 23.4% deliberate (suicidal), and the rest (76.6%) were unintentional due to drug intoxication or accidental poisoning. Oral poisoning and envenomation incidences were 93.1% and 6.7%, respectively. Two patients were poisoned due to inhaling the vapor of hydrochloric acid (HCl). Two patients were exposed to sulfuric acid. Also, there was one case of bleach intoxication. However, the most common substance from chemical products was organophosphates. There were 17 patients with poisoning from organophosphate pesticides. Most registered poisonings were at home (72.8%), following 7% at work, while 20.2% were unknown. There was a significant difference in terms of toxicant type (p < 0.001) and the cause of poisoning (p = 0.042) between men and women (Table 3).

**Table 3:** Comparison of toxicant type and cause of poisoning by gender.

Variable	Male	Female	Total	Test result
Toxicant type				
Pharmaceutical	94 (36.7%)	163 (58.6%)	257 (48.1%)	p < 0.001 (X^2^ = 32.208, df = 5)
Envenomation	25 (9.8%)	13 (4.7%)	38 (7.1%)	
Narcotics	82 (32.0%)	56 (20.1%)	138 (25.8%)	
Alcohol	8 (3.1%)	1 (0.4%)	9 (1.8%)	
Chemical	29 (11.3%)	25 (9.0%)	54 (10.1%)	
Unknown	18 (7.1%)	20 (7.2%)	38 (7.1%)	
Total	256 (100.0%)	278 (100.0%)	534 (100%)	
Cause of poisoning				
Suicide	50 (19.5%)	75 (27.0%)	125 (23.4%)	p = 0.042 (X^2^ = 4.123, df = 1)
Unintentional	206 (80.5%)	203 (73.0%)	409 (76.6%)	
Total	256 (100.0%)	278 (100.0%)	534 (100%)	

Table 4 shows a difference between toxicant type and the cause of poisoning (p < 0.001). Signs or symptoms among the poisoned patients and the percentage in all 534 cases are shown in Table 5. The five common discomforts revealed to be nausea (36.5%), unconsciousness (30.0%), vertigo (27.1%), vomiting (26.4%) and pain (22.5%). Regarding previous disorders, 18.2% of patients had a history of opioid addiction, and 10.8% had known psychiatric diseases.

**Table 4:** Comparison of toxicant type by cause of poisoning.

Variable	Suicide	Unintentional	Total	Test result
Toxicant type				
Pharmaceutical	88 (70.4%)	169 (41.3%)	257 (48.1%)	p < 0.001 (X^2^ = 39.530, df = 5)
Envenomation	0 (0%)	38 (9.3%)	38 (7.1%)	
Narcotics	20 (16.0%)	118 (28.8%)	138 (25.8%)	
Alcohol	0 (0%)	9 (2.3%)	9 (1.8%)	
Chemical	8 (6.4%)	46 (11.2%)	54 (10.1%)	
Unknown	9 (7.2%)	29 (7.1%)	38 (7.1%)	
Total	125 (100.0%)	409 (100.0%)	534 (100%)

**Table 5:** Signs/symptoms in studied poisoned patients; frequency distribution in 534 cases.

Entry	Disease history	Frequency distribution	Percentage
1	Nausea	195	36.5
2	Unconsciousness	160	30
3	Vertigo	145	27.1
4	Vomiting	141	26.4
5	Pain	120	22.5
6	Colic pain	78	14.6
7	Headache	44	8.2
8	Fatigue	30	5.6
9	Redness	27	5
10	Dyspnea	27	5
11	Burning	22	4.1
12	Swelling	18	3.4
13	Tremor	16	3
14	Tenderness	11	2
15	Delusion	8	1.5
16	Aggression	8	1.5
17	Tachycardia	7	1.3
18	Convulsions	7	1.3
19	Blurry vision	7	1.3
20	Agitation	5	0.9
21	Sweating	3	0.6
22	Ataxia	3	0.6
23	Lacrimation	2	0.4
24	Tinnitus	2	0.4
25	Chest pain	2	0.4
26	Photophobia	1	0.2
27	Itching	1	0.2
28	Heartburn	1	0.2
29	Hematochezia	1	0.2
30	Frequent urination	1	0.2

Prescribed or off-label pharmaceuticals included acetaminophen, alprazolam, aspirin, amoxicillin, chlordiazepoxide, chlorpromazine, clozapine, diazepam, dimenhydrinate, hydroxyzine, ibuprofen, mefenamic acid, methadone, nortriptyline, Novafen (Acetaminophen/Caffeine/Ibuprofen), oxazepam, phenytoin, ethanol, ranitidine, clonazepam, *Majoon* (hashish), topiramate, risperidone, amitriptyline, baclofen, nortriptyline, valproate, olanzapine, propranolol, diclofenac, diazepam, clidinium C, bupropion, levetiracetam, lithium, captopril, citalopram, sertraline, tramadol, adult cold, losartan, metronidazole, doxepin, cefixime, zolpidem and valsartan. Pill consumption among the patients ranged from one tablet to 62 tablets. Regarding opioids such as methadone and tramadol, 1 to 8 tablets were abused. Comparison of toxicant type by gender showed sex differences in taking narcotics and pharmaceutical products. Men used narcotics more than women (59.4%), while women had pharmaceutical poisoning more than men (63.4%). According to the medical records, one patient passed away in the current study, and the others were released from the hospital following treatment duration with full recovery or with personal consent. No further information was available concerning post-discharge outcomes (i.e., patients were not followed up after discharge from the hospital if they had developed delayed complications secondary to the acute poisoning). The deceased patient in the current study was a 34-year-old woman who was intoxicated after taking warfarin and alprazolam. She had intentionally taken an unknown amount of warfarin and alprazolam tablets. She was referred to the emergency department in an unconscious state. The treatment duration of this patient lasted for nine days.

## Discussion

In most cases of our study, toxicity was related to pharmaceutical products (48.1%), which were commonly administered orally (93.1%) at home (72.8%). The most common pharmaceuticals were benzodiazepines (BDZ), especially alprazolam (7.9%) and clonazepam (7.5%). Next, acetaminophen (9.9%) was the most common drug. The availability of alprazolam and clonazepam without prescription might be a major contributor to high BDZ use and subsequent intoxication. The main types of narcotics were opioids, especially opium and methadone. Most cases of cannabis abuse were in the form of taking a specific kind of cannabis, which is native to Birjand and called Majoon-Birjandi, or *Majoon*. Unlike marijuana, which is smoked, it is taken orally. However, it poses the same effects, including delusion, excessive laughter and increased appetite. Surprisingly, this region's studies even indicate the children's poisoning by *Majoon*^15^. In our study, methadone and tramadol poisoning cases illustrate that even a single tablet could be toxic. Considering this and that at least 10,000 cases of opioids poisoning occur annually in Iran, according to authorities, the government should enhance pharmacovigilance rules and ask pharmaceutical companies to update the relevant drug supply guidelines and decrease tablet dosage, especially in the production of methadone and tramadol. More medication surveillance can help prevent easy drug access and decrease poisoning and overdose. Another study evaluated the epidemiology and agents of poisoning in poisoned patients admitted to the referral intensive care unit in Birjand and found that pharmaceutical medication, opioids and pesticides were the most common exposures^16^. Shokrzadeh et al.^17^ also showed that poisoning cases were due to drug overdose. In Shokrzadeh et al. study, 227 out of 800 cases of hospitalized poisonings were related to non-pharmaceutical products^17^. Based on our findings, at least half of the patients had no previous history of diseases. Among patients with a previous medical history, addiction was the most common. Such a background might have an impact on the incidence and recurrence of more poisonings in the future. Among different poisonings, poisoning by narcotics accounted for 25.8% of all cases in the current study. Several studies in different regions of Iran showed that the most common toxicant type was narcotics. For instance, Farzaneh et al. in Ardabil (northwest of Iran)^18^, Afzali et al. in Hamedan (western Iran)^6^, and Ayatollahi et al. in Yazd (central Iran)^19^ showed drug abuse as the most common cause of acute intoxication. Iran is heavily involved in opioid abuse due to its shared border with Afghanistan^20^. Hassanian-Moghaddam et al. found that opium and methadone were the most common drugs of abuse in adults in Tehran. They revealed that narcotics toxicity contributed to 24.7% of deaths^21^. Apart from narcotics, some studies reported BDZ, tricyclic antidepressants^16^, psychoactive drugs^22^, antiepileptics and sedative-hypnotics^21^ as the leading agents of poisonings. Hovda et al. reported that BDZ, ethanol and paracetamol were the leading cause of toxicity among poisoned patients^23^.

As seen in Table 1, alcohol is a toxicant, and exposure may result in acute effects. Although ethyl alcohol can be toxic if used excessively, the deep concerns over drinking overdose are related to methanol poisoning. The possession, production and consumption of alcoholic beverages have been illegal in Iran since 1979. Since then, the governors have faced an uphill battle to cleanse the country of bootleg liquor. However, laws governing are being roundly ignored at weddings and parties. The non-factory production of alcoholic beverages might produce contaminated alcoholic products. As a result, after the consumption of contaminated liquors, there is a possibility of methanol poisoning. Sometimes, it causes mass methanol poisoning, referring many to the emergency department^24,25^. Following addiction, psychiatric disorders were common in our findings. 29% of patients had a history of addiction and psychiatric disorders. Since the risk of poisoning may be increased by such a history, it is important to monitor and track individuals with the background mentioned earlier. Dragisic et al. found that psychiatric history and addiction were the most common among patients who committed suicide^26^. Senanayake and Karalliedde showed that intentional poisonings in cases between 11 and 30 years old in Sri Lanka happened in people with a psychiatric disorder history^27^.

In our study, many patients required less than four days of treatment. The mean hospital stay was 3.0 ± 3.3 days for patients who attempted suicide, ranging from 1 to 19 days. The duration of hospitalization varied in different studies. Sing and Aacharya reported that hospital stay was 1–16 days^28^. Nair and Revi reported that the mean hospitalization period was 5.5 days^29^. Abubakar et al. showed that the duration of hospitalization for poisoning cases ranged from 1 to 41 days^30^. Among the recorded signs/symptoms in Table 5, redness and swelling were generally seen in the bitten individuals. Most envenomation cases in Birjand among the current study patients were by scorpion stings (77.8%, n = 28), followed by snake bites (11.1%, n = 4).

Moreover, one of the patients admitted with tramadol showed photophobia. Other complications in this patient were vomiting, vertigo, and nausea. Photophobia is not a common adverse effect of taking tramadol. However, it may happen that tramadol was prescribed for naive patients or females, as was the case in this study. While there was only one death from poisoning, the mortality rate was revealed to be 0.19% during this study. The mortality rate in some other studies was 0.3%^31^, 1.3%^32^, 7.8%^27^, 4.2^29^, and 16%^26^.

The results showed that most poisonings occurred in spring. This finding was contrary to the Jalali study in which most poisonings were in autumn^31^. This discrepancy might be due to the geographical diversity and the incidence of envenomation. Seasonal distribution of poisoning varies among different studies, and the incidence may occur at any time of the year. For example, most cases of poisoning in the Mehrpour et al. study and Shokrzadeh et al. study were in the summer^15,17^. The present study results also showed that the rate of poisoning in patients between 15 and 35 years was higher than in the other age groups, and intentional poisonings were noticeable (23.4%). Surprisingly (25%) of cases were students. Students may attempt suicide easier than adults with a higher frequency of adjustment disorder in this group. Suicide prevention programs for students should focus on the early identification of at-risk students and the appropriate treatment of episodes of psychopathology. For this age group, psychological factors are considered as the determinative indicator^33,34^. The inability to develop coping skills seems to be probable in young adults^35^. This finding was consistent with the findings of some other studies^28,32,36^. Based on related studies, findings generally indicate that the highest rate of intoxication occurs in adolescents and tragically happens among the active workforce^37,38^.

Significant gender differences were not observed in the number of referred poisoning cases to the hospital (p = 0.412). However, a female predominance in poisoning cases, similar to our findings, was reported by Lam et al.^39^. Although there was a significant difference between men and women (p = 0.042) regarding the cause of poisoning (Table 3), since the significance level was close to 0.05, such a difference might not be found in another population with a different sample size from our study. Moreover, the incidence of poisoning cases in married couples was higher than in the single population, which may be contrary to expectations. However, the geographic region seems to be associated with marital status, and as a result, there were more married cases of acute poisoning than single ones. Furthermore, intimate partner violence, sexual coercion and age disparity might be the underlying causes of such differences. Findings of studies of Shokrzadeh^17^, Nair and Revi^29^, and Guntheti^40^ confirm the present study in this regard.

Education is deterrent to the incidence of poisoning or suicide. We found that the incidence of poisoning was higher among patients without a college degree than among those with university education levels. Most patients were referred to the hospital a short time after the incident. In Jalali et al. study, 65.4% of poisoned patients were referred to a hospital less than 3 h after the incidence^31^. During the studied period, 74.6% of patients lived in urban areas, and the rest (25.4%) were in rural settings. This finding can be attributed to urbanization and the point that poisoned city residents are quickly or more frequently referred to a hospital. De Silva and Ratnayake also showed that drug poisonings were higher in urban areas^41^. Employment rates among poisoned patients were found to be 55.0% in our study. Noticeably, the poisoning rate was high among students (25.3%). The impact of romance issues, weak social connections and inability to use coping skills are probably the causes of intoxication in students who lie in adolescents and youth. Another study showed that students tend to attempt self-poisoning with pharmaceutical agents. In this study, the most common reason for the suicide attempt was family conflict followed by romantic issues. The most common psychiatric disorders among students who attempt suicide are adjustment disorder and major depression^18^. Therefore, the matter of intoxication in students should be seriously considered.

Other studies from our center have investigated this region's epidemiological and clinical profiles of poisoned patients. In a 7-year study in Birjand, pharmaceutical medication (36.6%) and opioids (26.2%) were the most common exposures in acute poisoning patients. The rate of suicide attempts (38.2%) was higher than in our study. During the study period, the mortality rate was 19.5%, much higher than our findings^16^. Another study in this region showed that the mean age of patients with illicit drug use was 34 ± 10.2 years. Contrary to our findings, a male predominance in poisoning individuals was observed with a ratio of 9.3/1. They found that 90.6% were related to the inhabitants of urban areas, similar to the current study^42^.

Our study had some limitations. Recruitment and data collection was limited to the data from a single treatment center, although the studied center is the province's main referral hospital for poisoning. The design of our study was another limitation. Some of the medical records were only partially registered because of the retrospective nature of this investigation. We only realized this issue at the end of the study when the patient history was under evaluation. Furthermore, no data were available concerning the outcomes after hospital discharge. Another limitation was that the Poisoning Severity Score (PSS) was not evaluated for our poisoning cases due to a contrary opinion about the reliability and validity of this score in the literature.

## Conclusion

Pharmaceutical product, such as methadone, alprazolam, clonazepam and acetaminophen, were our study's most common causes of poisoning. These drugs are more commonly to be taken orally and at home. In order to ensure that drug distribution and toxicants are controlled rigorously, pharmacovigilance regulation is necessary. A high rate of deliberate poisonings, particularly among young adults and especially among students, should be considered an urgent medical, psychological and social concern. A special focus should be placed on improving mental health and preventing substance use to decrease suicide attempts and overdoses.

## Data Availability

The datasets generated and analyzed during the current study are available from the first author, KN, on request. The dataset contains information on the age, sex, and location of the patients that are publicly sharing could compromise the privacy of research participants.
